# Dental Microwear Texture Analysis of the Bornean Orangutans ( *Pongo pygmaeus* ) From the Selenka Collection

**DOI:** 10.1002/ajpa.70266

**Published:** 2026-05-05

**Authors:** Sophie Gabriele Habinger, Gildas Merceron, Anneke H. van Heteren, Valeria Rojas Cuyutupa, Hervé Bocherens, Olivier Chavasseau

**Affiliations:** ^1^ Laboratoire PALEVOPRIM, UMR CNRS 7262 Université de Poitiers Poitiers France; ^2^ Department of Geosciences Eberhard Karls Universität Tübingen Tübingen Germany; ^3^ Universität Konstanz Konstanz Germany; ^4^ Zoologische Staatssammlung München Staatliche Naturkundliche Sammlungen Bayerns München München Germany; ^5^ GeoBio‐Center Ludwig‐Maximilians‐Universität München München Germany; ^6^ Department Biologie II Ludwig‐Maximilians‐Universität München Planegg‐Martinsried Germany; ^7^ Senckenberg Centre for Human Evolution and Palaeoenvironment At the University of Tübingen Tübingen Germany

**Keywords:** Borneo, dietary ecology, dietary reconstruction, Ponginae, Selenka orangutan collection

## Abstract

**Objectives:**

Characterizing the diet of extant taxa is important not only to determine their ecological niche but also to serve as a reference for dietary and niche inferences in evolutionary studies. Tracking the diets of fossil taxa and their change through time has been increasingly employed to further understand the evolution of primates. In the last decades, several studies using stable isotope analysis, dental topography, or dental microwear analysis have been conducted to reconstruct the paleoecology and diet of fossil pongines. However, paleodietary reconstructions based on the dental microwear of fossil pongines lacked a well‐defined and extensive reference dataset of extant *Pongo*.

**Material and Methods:**

To close this gap, we characterized intrapopulation variation in dietary ecology using dental microwear textures of 89 orangutans collected by Emil and Margarethe Selenka on Borneo in 1894.

**Results:**

Our study provides insights into dietary variation in extant *Pongo* and aims as an important reference dataset in future paleodietary reconstructions. According to climatic records available for the late 19th century, the specimens were not collected during an El Niño event; these events impact fruit resource abundance in Southeast Asia. We found no significant differences in dental microwear textures depending on sex, age, or locality.

**Discussion:**

This suggests that, despite a large number of specimens and localities sampled, sex, age, and locality did not significantly influence the dietary resources consumed at the scale of the population. More detailed individual information—not available for this historical collection—would be necessary to further test these results.

## 1 Introduction

Diet is an important component of the realized ecological niche of primates. For this reason, dietary characterization has been the focus of numerous ecological and evolutionary studies as well as studies of conservation led by primatologists and paleoprimatologists. Among the various methods employed to track the dietary preferences of primates, some are only applicable on living taxa (feces analysis, direct observation, etc.) while others can be applied on both fossil and living species, such as dental topography analysis (e.g., Berthaume et al. 2020; Merceron et al. 2006), chewing biomechanics (e.g., Marcé‐Nogué et al. 2017; Taylor 2006), dental stable isotope analysis (e.g., Habinger et al. 2022; Schoeninger et al. 1999; Sponheimer et al. 2006), and dental microwear analysis (e.g., Merceron et al. 2006, 2021; Scott et al. 2012; Thiery et al. 2024). The latter methods, which track the diet at different time resolutions (ranging from days to the macroevolutionary scale) are thus especially useful for evolutionary studies by providing a common basis for dietary characterization between fossil and modern data. In particular, these methods allow characterizing the diet of fossil primates with modern species as a reference.

The orangutans (Ponginae) have a fossil record that extends back to the Middle Miocene (Chaimanee et al. 2003; Kelley et al. 2025). This group, which became diversified (with genera *Gigantopithecus*, *Khoratpithecus*, *Sivapithecus*, *Indopithecus*, and *Ankarapithecus*) occupied during the Neogene a continental scale geographical range from Anatolia to Southeast Asia and China (Chaimanee and Jaeger 2023; Kappelman et al. 2003; Kelley et al. 2025). The extant genus *Pongo* is first recorded in the Early Pleistocene of China and presented a continuous geographical range from South China to Indonesia during the Pleistocene (e.g., Tshen 2016). The shrinkage of pongine distribution into Southeast Asia coincides with changes to dryer climates and fragmentation of forested habitats after the Middle Miocene Climatic Optimum (Badgley et al. 2008; Cannon et al. 2009; Habinger et al. 2022; Nelson 2007). From such a perspective, one could consider extant orangutans as refugee species. In contrast to the limited fossil record of other great apes (McBrearty and Jablonski 2005; Almécija et al. 2021), the extensive Neogene fossil record of pongines may provide very useful insight into their evolutionary ecology (e.g., Habinger et al. 2022). However, although recent contributions on the diet of modern species of *Pongo* have been published (e.g., Fiorenza et al. 2022, 2024), extensive comparative data sets are still needed to improve dietary characterizations of fossil pongines.

Modern orangutans (*Pongo*) are subdivided into three species. *P. abelii* (Sumatran orangutan) and *P. pygmaeus* (Bornean orangutan), were formerly considered as subspecies and are now recognized as distinct species (Groves 2001). A third species, *P. tapanuliensis* was more recently described from Sumatra (Nater et al. 2017). In Borneo, *Pongo pygmaeus* is subdivided into three subspecies. *P. p. pygmaeus* is present in northwestern Borneo (Sarawak and Kalimantan), nearly exclusively north of the Kapuas River, while *P. p. wurmbii* is present in Central and West Kalimantan, and *P. p. morio* is present in East Kalimantan and Sabah (e.g., Nater et al. 2017; Taylor 2006; Wich et al. 2008).

Orangutans are large apes with a low reproduction rate. They have the most arboreal habits among living hominids, using extensively suspensorial modes of locomotion. Bornean orangutans live in peat swamps and lowland primary or secondary dipterocarp tropical rain forests (Groves 1971; Strobel 2013) while Sumatran orangutans live predominantly in primary lowland tropical forests (Rijksen 1978; Urban 2008) and *P. tapanuliensis* inhabits rainforests (Laurance et al. 2020). Based on field observations, orangutans are described as very opportunistic feeders that use a large variety of food sources (e.g., Galdikas 1978; MacKinnon 1974), with fruits representing more than 50% of the diet (Galdikas 1988). The composition of their diet varies markedly from month to month. Fruits represent up to 90% in July and August but about 20% from February to May (MacKinnon 1974). Most of these fruits are figs, which are multiseeded but soft. Leaves and insects (and about 30% lianas and bark when fruits are not available) complete the diet (Galdikas 1988; Kanamori et al. 2010; MacKinnon 1974; Rodman 1977, 1988; Ungar 1995; Vogel et al. 2015, 2017). Dietary ecology differences between *Pongo* species and *Pongo pygmaeus* subspecies have been documented. For instance, *Pongo abelii* spends more time feeding on fruit than *P. pygmaeus*, which feeds on more diverse types of foods (see the compilation of Taylor 2006). Some dietary differences in the proportion of fruit and vegetation exist between *P. p. morio* and *P. p. wurmbii* while the dietary ecology of *P. p. pygmaeus* is poorly‐known (Taylor 2006). The diet of *P. pygmaeus* is affected by mast and peak fruiting periods. Contrary to the situation observed during nonfruiting periods, Bornean orangutans feed almost exclusively on fruit during a mast fruiting event (see Kanamori et al. 2010, 2017 for *P. p. morio*). Depending on resource availability, *P. pygmaeus* shows variation in foraging strategies among populations or age‐sex classes (Vogel et al. 2017). Male–female differences in terms of dietary ecology have been noted in several studies on *P. pygmaeus* . Adult males travel farther per day, resulting in a larger home range covered in the course of their lives in conjunction with an increased time spent on the ground. This behavior could result in a higher incorporation of termites and other terrestrial food sources instead of bark or young leaves found higher up in the canopy (Galdikas 1978, 1988; Rodman 1977, 1988). As a consequence of their predominantly frugivorous diet, orangutans act as important fruit dispersers that influence the regeneration and distribution of plants, and are essential for natural forest dynamics (McConkey 2018; Tarszisz et al. 2018). A recent study suggests that the Ponginae may have been important fruit dispersers since the late Miocene (Spengler et al. 2023).

The earliest work on molar microwear analysis on extant orangutans and one of its fossil relatives (*Sivapithecus*) was performed as part of a comparative study of different primate taxa (Teaford and Walker 1984). Another study of the incisor microwear of Sumatran anthropoid primates (including *P. abelii* ) also yielded insight into diet and tooth use (Ungar 1994). Subsequently, a comparative dietary study of the hominoid *Griphopithecus alpani* based on a microwear analysis included about 20 specimens of *Pongo pygmaeus* (King et al. 1999). Another molar microwear study on *Ouranopithecus macedoniensis*, a late Miocene hominoid, included 56 individuals of orangutans as a comparative baseline (Merceron et al. 2005). Forty‐two of them actually belong to the Selenka collection, which is the focus of the present study. The study of Merceron et al. (2005), similarly to all of those cited above, was however based on a 2D microwear analysis, a methodology characterized by poor repeatability and low interest in regards to open science practices (Mihlbachler et al. 2012).

The first 3D dental microwear texture analysis including extant orangutans ( *P. pygmaeus* , *n* = 7) was dedicated to the diet of *Khoratpithecus* (Merceron et al. 2006), a late Miocene hominoid whose several lines of evidence suggest that it is the sister‐group to *Pongo* (e.g., Chaimanee et al. 2019, 2022; Pugh 2022). The authors concluded that *Khoratpithecus* was predominantly frugivorous with no evidence of hard or tough food items in the diet (Merceron et al. 2006). Orangutans were also analyzed as part of a study attempting to establish a comparative baseline for DMTA across 21 anthropoid primate species including 15 individuals of *P. pygmaeus* (Scott et al. 2012). These data were then used recently in two investigations of the diet of Pleistocene *Pongo* from Sumatra (augmented by 12 modern specimens of *P*. *pygmaeus*; Louys et al. 2021) and Southern China (Fan et al. 2024). A comparison between modern and fossil data indicated that Pleistocene *Pongo* from Sumatra consumed less tough food (e.g., lianas, bark, and mature leaves) than the modern individuals from Borneo (Louys et al. 2021). This difference corresponds to observations of dietary differences between *P. abelii* and *P. pygmaeus* today (Louys et al. 2021).

Here, we characterize the diet of a wild orangutan population ( *Pongo pygmaeus* ) hunted in Borneo (West Kalimantan) using dental microwear texture analysis (DMTA), a method known to detect inter‐individual variations in diet (see for instance Berlioz et al. (2017) for cases on present‐day cervids and Percher et al. (2018) for case studies on present‐day primates). We analyzed the dental microwear textures of 89 individuals collected during an expedition to Borneo in 1894 and deposited as the Selenka collection housed at the Zoologische Staatssammlung München (ZSM), Germany. Although there were definite human‐orangutan interactions as early as around 50 ka, humans did not have a significant impact on orangutan populations before 3 ka (Spehar et al. 2018). Even at the end of the 19th century, the anthropogenic destruction and fragmentation of orangutan habitat was less prevalent than it is now. The Selenka collection can then serve as a robust comparative dataset for palaeoecological reconstructions of fossil pongines and early hominids.

Our study on *Pongo pygmaeus* offers the possibility to detect dietary variations due to (i) differences between sex, (ii) age class, (iii) spatial variations, or (iv) seasonal food availability, notably during mast fruiting periods. We use historical precipitation records and indices modeling the ENSO cycle (El Niño Southern Oscillation), which is heavily linked to mast fruiting (Williamson and Ickes 2002), to assess if mast fruiting events might have occurred in the years the Selenka *Pongo* were collected.

## 2 Methods

### 2.1 The Selenka *Pongo* Collection and the Origin of the Specimens

**FIGURE 1:**
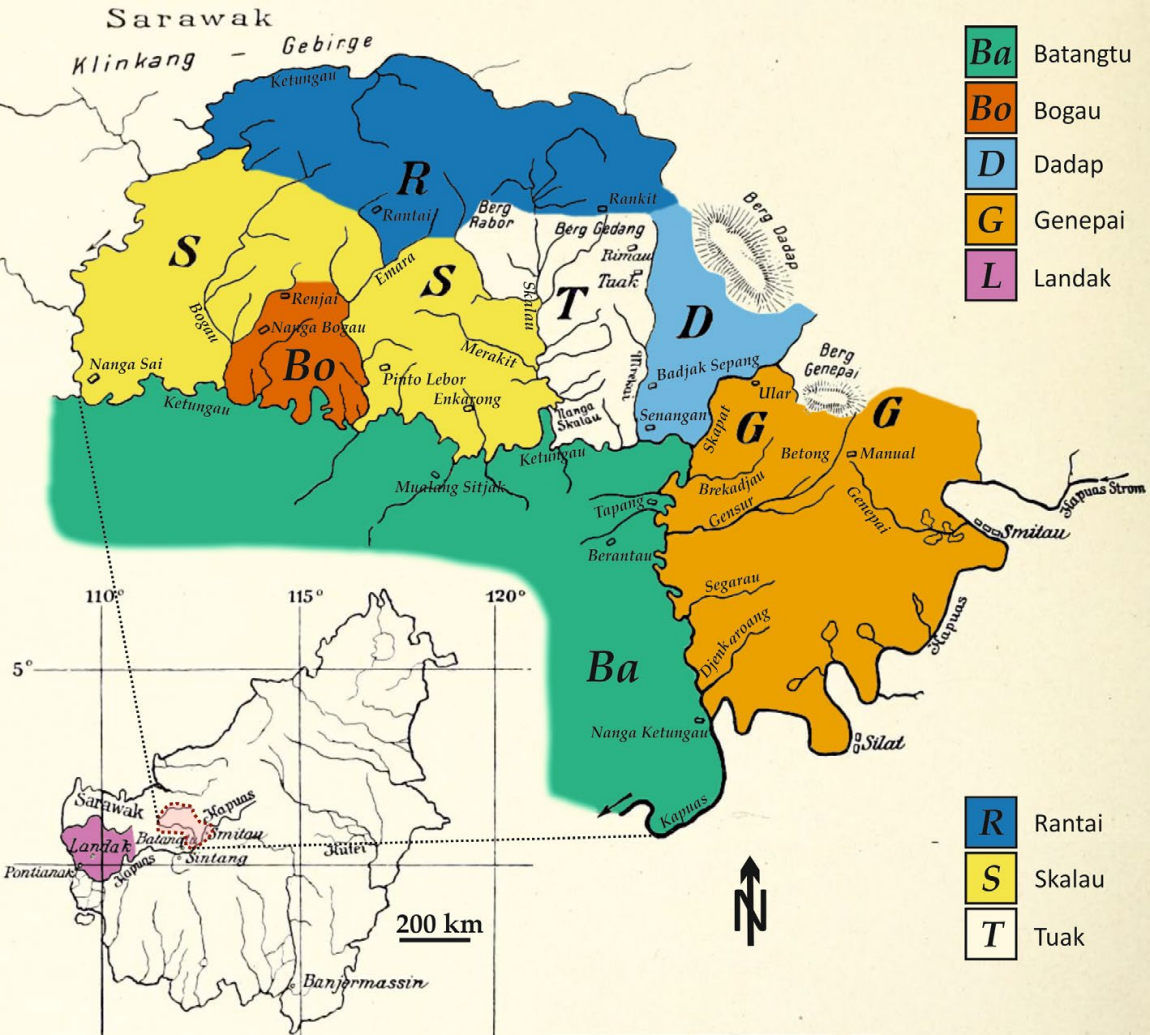
Map showing the areas of provenience of the Borneo orangutan ( *Pongo pygmaeus* ) specimens from the Selenka collection (after Selenka 1898). The areas were delimited based on the descriptions made by Selenka and on natural barriers (main rivers and mountains). The Bogau area was not present on the original map but was added around the village of Nanga Bogau because this indication of provenience appears on the original labeling of some of the specimens.

The Selenka collection comprises around 300 orangutan skulls across all age groups from the West Kalimantan province, on Borneo (Selenka 1896, 1898; see also Supporting Information for historical data on this collection). On the map (Figure 1), different areas are marked, which Selenka correlated with local morphotypes. We use Selenka's labels solely as an indication of their point of origin. Although the Bogau area was not correlated to a morphotype, he used the label on some of the orangutan skulls. We therefore added it to the map using the location of the village Nanga Bogau as a point of reference.

The habitat north of the Kapuas River is a mixed dipterocarp forest, with peat swamp forests only present in the most southwest parts of the Landak region close to the coast (Phillips 1998). The range of altitudes of the collection area is low, usually below 300 m, with the exceptions of four mounts (“Berg Genepai” = probably Gunung Kenepai, 1112 m; “Berg Dadap” = probably Gunung Tutoop, 805 m; “Berg Gedang” = probably an unnamed peak of 835 m; “Berg Rabor” = probably Gunung Kehuma, 1210 m). Considering their provenience (north of the Kapuas River), the specimens of the Selenka collection probably correspond to the subspecies *Pongo pygmaeus pygmaeus* , a subspecies for which, interestingly, few dietary information is available (Taylor 2006).

We studied dental microwear texture of 89 wild *Pongo pygmaeus* individuals from the Selenka collection (Tables 1 and S1). All specimens are housed in the Zoologische Staatssammlung München, Germany (SNSB‐ZSM). We selected in priority specimens with worn second molars, excluding the youngest specimens as well as senile individuals for which occlusal molars surface is too much worn. We discarded specimens with too much organic and inorganic deposits on enamel surface to limit the cleaning work and maximize the probability of sampling an unbiased biological signal. From each specimen we analyzed one grinding (f9) and one shearing facet (f6) of a second molar (Maier and Schneck 1981). The second molars are preferred for tooth wear analysis, as they display intermediate tooth wear between first and third molars, the former being often too worn and the latter being too lightly worn on adult specimens. We used the age class system and age assignments of the SNSB‐ZSM database. In our sample, the juvenile specimens correspond to individuals with only lightly worn lower m2s. The subadults present more worn m2s and erupting m3s or erupted but unworn m3s. Adults show molar wear on m3s. Although there is no statistically significant difference in microwear pattern on homologous dental facets between upper and lower molars (Teaford and Walker 1984), we chose to sample the lower second molar whenever possible to maximize sample homogeneity. In the case of the Selenka sample females are far more numerous than males. All of the locations in Figure 1 except for Tuak are represented in our dataset.

**TABLE 1:** Summary of the number of individuals of *Pongo pygmaeus* from Borneo for each age and sex group from which f9 and f6 were analyzed.

Sex	Female				Male		
Age	Juvenile	Subadult	Adult	Senile	Juvenile	Subadult	Adult
*Pongo pygmaeus*	5	4	45	1	1	6	27

### 2.2 Dental Microwear Textural Analysis

Dental microwear provides direct evidence of an animal's diet during the last days to weeks of an animal's life. This is known as the “last supper” effect (Grine 1986). The turnover rate of dental microwear signal depends on food mechanical properties with harder/stiffer foods leading to a more rapid turnover than softer/ductile food items (Teaford and Oyen 1989; Teaford et al. 2017, 2021; Winkler et al. 2020). As a result, dental microwear texture analysis tracks seasonal variations in diet (Berlioz et al. 2017; Merceron et al. 2010; Percher et al. 2018; Teaford and Robinson 1989).

Dental microwear texture analysis (DMTA) is a 3D method able to characterize the texture created on wear facets of the occlusal surface of teeth during mastication. The method has been proven to reduce inter‐ and intra‐observer errors compared to former 2D methods based on visual counting, measurements, and classifications through stereomicroscopes or computer screens (DeSantis et al. 2013; Galbany et al. 2005; Grine et al. 2002; Mihlbachler et al. 2012). DMTA is therefore more objective and produces more repeatable results (Scott et al. 2006).

#### 2.2.1 Preparation and Scanning of Molds

To remove any dirt or coatings used for preservation, we cleaned the teeth with ethanol using cotton swabs before molding. We then took silicone molds of the teeth (polyvinylsiloxane addition‐type, Coltène‐Whaledent). Specific wear facets were scanned with a Leica DCM 8 white‐light scanning confocal microscope with a ×100 objective. We chose facets that represented both phase I shearing facets (f6) and phase II grinding facets (f9) (Maier and Schneck 1981). The scanned surfaces were imported in the LeicaMap v. 8.0 software (Mountain technology, Leica microsystems) and treated following a previously published protocol (Merceron et al. 2016; see also Supporting Information). Then, a 200 × 200 μm grid was extracted on which the textural parameters are calculated. Photosimulations and false color maps of all scans used in this study are attached as Supporting Information.

#### 2.2.2 Textural Parameters

Scale sensitive fractal analysis (SSFA) parameters (Scott et al. 2006) were calculated (see Table S1). We considered complexity (Asfc), anisotropy (epLsar), and heterogeneity of complexity calculated with 9 and 81 cells (HAsfc9 and HAsfc81, respectively; Table 2). Complexity is associated with the hardness of food items, with harder items or a larger proportion of harder items in an organism's diet leading to higher Asfc values. Overlapping pits and scratches of varying sizes and depths characterize these types of highly complex surfaces. Anisotropy is an indication of food toughness and shows increasing values with structures that share a similar orientation such as parallel striations. The heterogeneity of the complexity describes variations of the complexity across a surface (see Scott et al. (2006) for detailed description; see also Supporting Information). Additionally, we computed surface texture analysis (STA) parameters following well‐established procedures (Kaiser et al. 2016; Schulz et al. 2013) using the trident software (Thiery et al. 2024) and modified parameters following recommendations of Francisco et al. (2018). The combination of both SSFA and STA parameters allows for a comprehensive representation of the surface textural information (Calandra et al. 2012). For STA, we focused on seven height parameters, two surface parameters, and two volume ones (Table 2; Calandra et al. 2012; Kaiser et al. 2016; Schulz et al. 2013).

**TABLE 2:** Description of the scale sensitive fractal analysis (SSFA) and surface texture analysis (STA) parameters used in this study.

Abbreviation	Parameter type	Description	Unit
Asfc	SSFA	Complexity, area‐scale fractal complexity	—
epLSar	SSFA	Anisotropy, exact proportion length‐scale anisotropy of relief	—
HAsfc9	SSFA	Heterogeneity of complexity, surface subdivided into 3 × 3 subsurfaces	—
HAsfc81	SSFA	Heterogeneity of complexity, surface subdivided into 9 × 9 subsurfaces	
Sq	STA	Standard deviation of the height distribution, or root mean square (RMS) surface roughness	μm
Ssk	STA	Skewness of the height distribution	—
Sku	STA	Kurtosis of the height distribution	—
Sp	STA	Maximum peak height, height between the highest peak and the mean plane	μm
Sv	STA	Maximum pit height, height between the mean plane and the deepest valley	μm
Sz	STA	Maximum height, height between the highest peak and the deepest valley	μm
Sa	STA	Arithmetic mean height or mean surface roughness	μm
Sal2_0.5	STA	Auto‐correlation length (*s* = 0.5)	μm
Str2_0.5	STA	Texture aspect ratio (*s* = 0.5)	—
metf	STA	Mean depth of furrows	μm
medf	STA	Mean density of furrows	cm/cm^2^

*Note:* STA parameters are indicated based on ISO/FDIS 25178 norm.

#### 2.2.3 Statistical Analysis

As the analysis is run on two types of dental facets per individual, the number of variables is high. To reduce dimensionality of the data and to identify the most contributing parameters, we ran two principal component analyses (PCA) including all the *P. pygmaeus* individuals and all parameters described in Table 2. The detailed results of PCA are reported in Table S1 and Figures S1 and S2. We used Statistica (version 14.0.1.25) for the PCA. We also used Statistica to run ANOVAs on the obtained PC scores to test the statistical significance of the locality factor on adult specimens and the sex factor on the sample of adult specimens from Skalau locality, the only one whose sample size allows us to test the hypothesis of sexual differences in dental microwear (see Table 3).

**TABLE 3:** Results of the ANOVAs run on the rank transformed data on the first five dimensions of the PCA of all *Pongo pygmaeus* adult individuals.

Dimensions	df	SS	MS	*F*	*p*
A: ANOVA testing differences between localities					
DIM 1					
Intercept	1	26,558.49	26,558.49	127.52	< 0.001
Sex	1	11.23	11.23	0.05	0.817
Error	47	9788.77	208.27		
Total	48	9800.00			
DIM 2					
Intercept	1	28,952.62	28,952.62	143.26	< 0.001
Sex	1	301.52	301.52	1.49	0.228
Error	47	9498.48	202.10		
Total	48	9800.00			
DIM 3					
Intercept	1	27,217.04	27,217.04	130.61	< 0.001
Sex	1	5.94	5.94	0.03	0.867
Error	47	9794.06	208.38		
Total	48	9800.00			
DIM 4					
Intercept	1	26,800.18	26,800.18	128.55	< 0.001
Sex	1	1.48	1.48	0.01	0.933
Error	47	9798.52	208.48		
Total	48	9800.00			
DIM 5					
Intercept	1	27,008.21	27,008.21	129.53	< 0.001
Sex	1	0.37	0.37	0.002	0.966
Error	47	9799.63	208.50		
Total	48	9800.00			
B: ANOVA testing differences between males and females from the Skalau locality (only locality with sufficient sample size in both sexes)					
DIM 1					
Intercept	1	40,238.78	40,238.78	62.98	< 0.001
Locality	7	6989.26	998.47	1.56	0.158
Error	81	51,750.74	638.90		
Total	88	58,740.00			
DIM 2					
Intercept	1	39,752.17	39,752.17	56.49	< 0.001
Locality	7	1743.85	249.12	0.35	0.925
Error	81	56,996.15	703.66		
Total	88	58,740.00			
DIM 3					
Intercept	1	46,978.07	46,978.07	71.77	< 0.001
Locality	7	5720.77	817.25	1.24	0.286
Error	81	53,019.23	654.56		
Total	88	58,740.00			
DIM 4					
Intercept	1	28,070.34	28,070.34	41.07	< 0.001
Locality	7	3388.08	484.01	0.70	0.665
Error	81	55,351.92	683.36		
Total	88	58,740.00			
DIM 5					
Intercept	1	28,914.40	28,914.40	43.19	< 0.001
Locality	7	4518.56	645.51	0.96	0.462
Error	81	54,221.44	669.40		
Total	88	58,740.00			

Abbreviations: df, degrees of freedom; *F*, *F* ratio/test statistic; MS, mean squares; SS, sum of squares.

For the SSFA parameters we plotted the variation per sex, sex‐age, and locality group for both facets. We report boxplots for SSFA parameters on sexes, sex‐age groups and localities on both facets in Figures S3–S5. Data plots were created with the ggplot2 package (version 3.3.6).

## 3 Results

**FIGURE 2:**
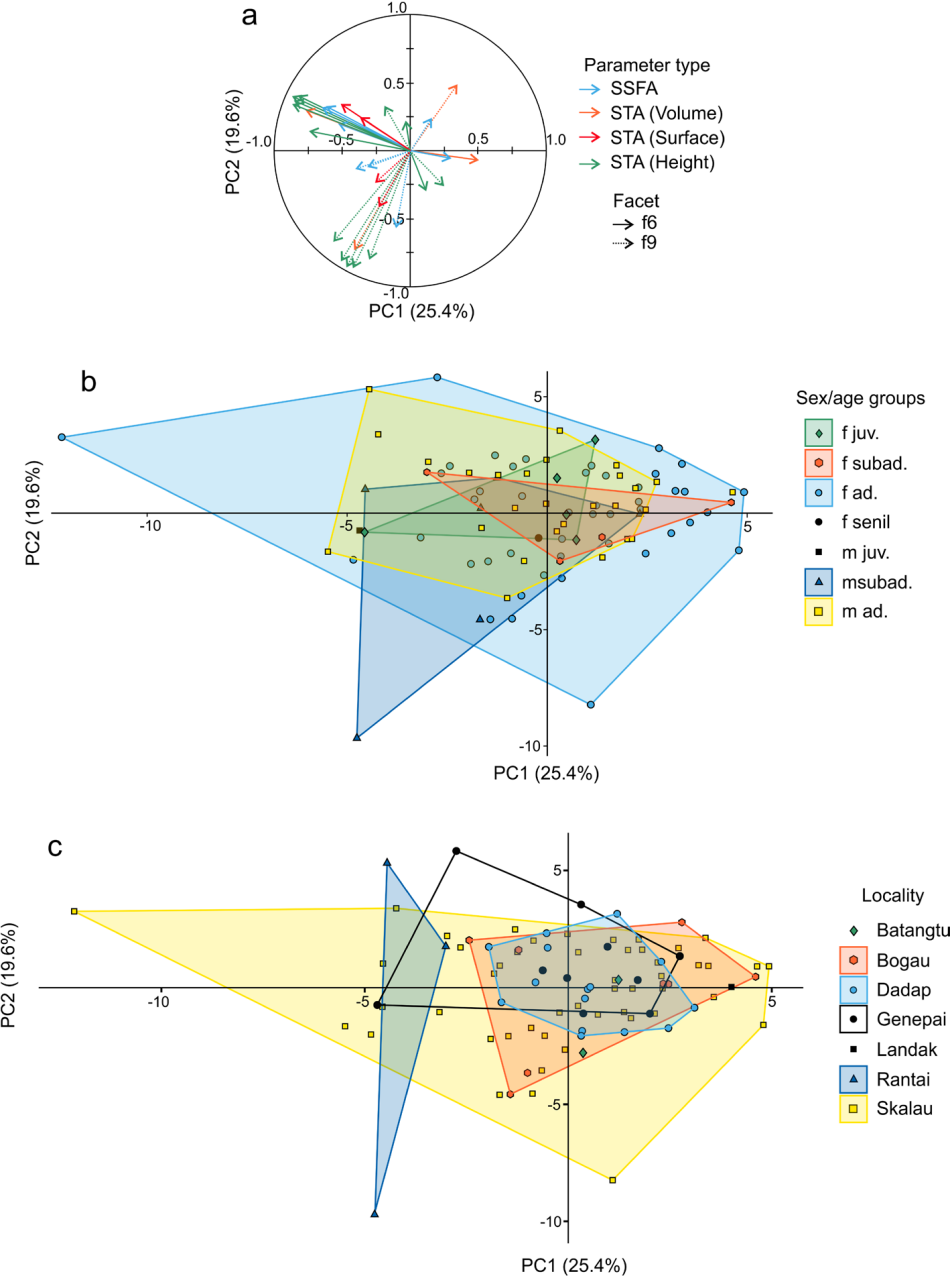
Correlation circle (a) and distributions of *Pongo pygmaeus* individuals from Borneo along PC1 and PC2 of the different sex‐age groups (b) and localities (c). Full details about the percentage of explained variance from PC1 to PC10 and the contributions of STA and SSFA parameters on PC1 and PC2 are available in Figure S1.

Focusing on the PCA, PC1 accounts for 25.4% of the total variance, the second PC for 19.6% (Figure 2). There is a marked drop after this with PC3 and PC4 accounting for less than 10% of the total variance each (Figure S2). As all the parameters of crushing (f9) and shearing (f6) facets were combined in the analysis, the most contributing variables along PC1 are height, volume, complexity, and heterogeneity of complexity parameters of the shearing facet. PC2 mostly reflects variability in height, volume, and general surface complexity parameters of the crushing facet. PC1 is negatively correlated with all of the STA and SSFA parameters on both facets except for anisotropy (epLsar), mean density of furrows (medf), and the skewness of the height distribution (Ssk). PC2 is negatively correlated with all of the STA and SSFA parameters of the crushing facet except for anisotropy (epLsar) and mean density of furrows (medf). PC2 is, however, positively correlated with the STA and SSFA parameters of the shearing facet except for anisotropy (epLsar) and mean density of furrows (medf).

Looking at individual distribution, there are no visible differences in DMTA pattern between males and females (Figure S2). Looking at age and sex‐age groups, adults show a higher variability than the subadults and juveniles (Figure 2b), most notably for the female specimens. However, this result is most probably caused by the sample characteristics in which female adults are the most abundant group. All the localities have very similar DMTA patterns (Figure 2c). Regarding locality and sex factors, we confirmed the absence of differences in DMTA pattern based on the PCA results with ANOVAs run on all the PC scores (Table 3) after rank‐transformation (Conover and Iman 1981).

**FIGURE 3:**
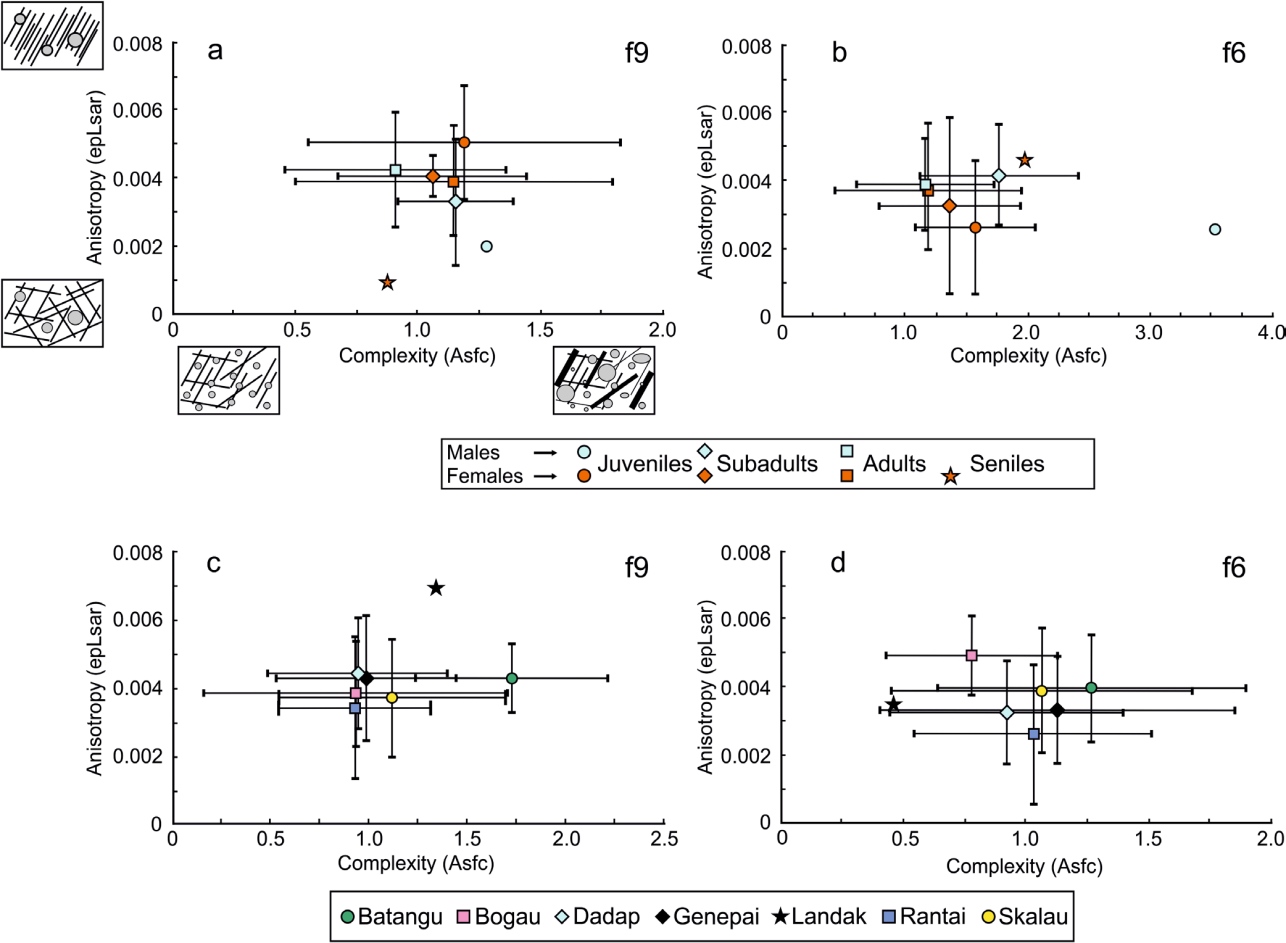
Plots of mean complexity and anisotropy values ±1 SD of the *Pongo pygmaeus* individuals from Borneo. (a and b) Individuals grouped by sex and age. (a) Grinding facet 9. (b) Shearing facet 6. (c and d) Individuals grouped by locality. (c) Grinding facet 9. (d) Shearing facet 6.

There are some interesting patterns if we only focus on the SSFA parameters. We looked at complexity (Asfc), anisotropy (epLsar), and heterogeneity (HAsfc) for each of the two facets. There was no difference in any of these parameters between males and females across all age groups. However, when looking at sex‐age and location groups, some tendencies were visible. When we looked at the different locations, it appears that the anisotropy and complexity values are quite homogeneous (Figure 3). The only notable exception is the Asfc values on the crushing facet f9 of the Batangtu individuals, which are higher than those of all other localities (Figure 3).

**FIGURE 4:**
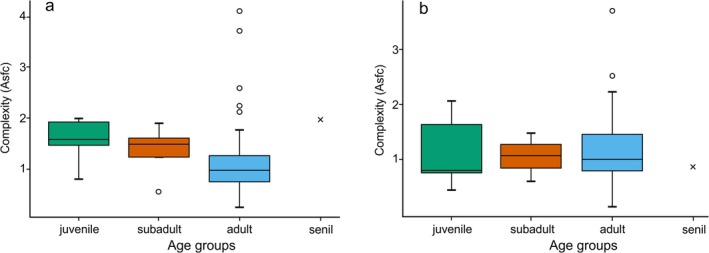
Boxplots of complexity (Asfc) on the shearing facet (a) and grinding facet (b) of the *Pongo pygmaeus* female individuals from Borneo sorted by age groups. The line in each box represents the median of the distribution. Circles represent outliers. Crosses represent single individuals.

There are some tendencies concerning the complexity values (Asfc) through age. In female individuals, complexity decreases with age class on the shearing facet but remains stable on the grinding facet (Figures 4 and S4). The tendency on Asfc.f6 is, however, nonsignificant (one‐way ANOVA on the age factor after data rank transformation: df = 3, *F* = 1.92, *p* = 0.138). Adult males also show lower complexity values than subadult males on the shearing facet (Figure S4). However, contrary to females, the complexity values of adult males are slightly lower than those of subadult males on the grinding facet (Figure S4). Boxplots on all three SSFA parameters (Asfc, epLsar, and HAsfc81) can be found in Figures S3–S5.

## 4 Discussion

### 4.1 Dietary Variation in *Pongo pygmaeus*

The obtained microwear data for *P. pygmaeus* are extremely homogeneous and show overall that no major difference between sex, age, and locality can be demonstrated in the Selenka collection. Part of the dental microwear inter‐individual variation we could observe in the Selenka *Pongo* collection might be caused by seasonal differences in diet or by spatial variations in resource availability. Unfortunately, we do not have precise information about the individual date of death, and thus, the effect of season cannot be explored. However, some historical records suggest that Selenka and his team were not actively hunting during the rainiest months because of limited field accessibility. Hence, it is likely that the seasonal effect on microwear signal is reduced. Two recent studies investigated the dietary ecology of *Pongo* through its macrowear pattern, which reflects the dietary adaptations of an animal (Fiorenza et al. 2022, 2024). Among extant hominids, the macrowear patterns of *Pongo* differ from those of *Pan* but shows similarities with those of *Gorilla*, although gorillas have different diets than orangutans (Fiorenza et al. 2022). The similarity of macrowear patterns between *Pongo* and *Gorilla* may be caused by the fact that the molar occlusal morphology is poorly constrained by soft foods (e.g., soft fruit). A study on *Pongo* molar macrowear including *P. abelii* and the three subspecies of *P. pygmaeus* (Fiorenza et al. 2024) found no statistical difference between these two species of *Pongo*, reflecting a shared frugivorous diet and pinpointing to the antiquity of their adaptative molar crown features. The same study found no statistical difference between males and females of *P. pygmaeus* , similarly to our microwear study on *P. p. pygmaeus*. It should be noted, however, that these results have different implications since macrowear reflects long term dietary adaption contrary to microwear. Thus, the hypothesis of dietary difference between male and female orangutans, which is not supported by either DMTA or macrowear, needs to be further tested on other populations (especially from *P. p. morio* and *P. p. wurmbii*). A few macrowear variables are different between *P. p. wurmbii* and *P. p. pygmaeus*, which partially corroborates formerly noted differences in mandibular functional anatomy (Taylor 2006). Thus, large‐scale spatial differences in terms of dietary ecology seem to exist in *Pongo pygmaeus* but are only documented for dental proxies by macrowear at the subspecies level. Our geographically and taxonomically more restricted study does not allow recognizing such dietary differences. Nevertheless, this does not mean that applying DMTA on a broader sample of *P. pygmaeus* would not reveal geographically different dietary patterns. DMTA analyses of *Pongo pygmaeus* have been performed in two studies (Louys et al. 2021; Scott et al. 2012), which give access to the microwear patterns of 27 individuals in total. Unfortunately, in this sample, no detailed provenience for most specimens (provenience known for only four specimens; Louys et al. 2021) or precise taxonomic status information (e.g., subspecies) are available. In addition, these two studies focused on grinding facets microwear patterns (e.g., facets 9, *x*, 10*n*) but without any information about the tooth selected, the facet analyzed, the sex or the age class for each specimen. Hence, the possibilities of comparisons between the results of these studies and ours are necessarily very limited. If we restrain our comparisons to the grinding facets, we observe at first glance that the range of complexity values are similar, being mostly comprised between ~0.2 and ~2.0 with some individuals displaying higher values (up to 4). However, in terms of proportions, only 8% of Selenka specimens have complexities > 2 against ~30% in the sample of Louys et al. (2021) and Scott et al. (2012). Concerning anisotropy values, the Selenka sample displays a greater range of values (~0–0.0075) than in the sample of comparison (~0.001–0.0065). The proportions of individuals with anisotropies > 0.005 is 27% in our Selenka collection sample against 33% in that compiled in Louys et al. (2021). Thus, it seems that the two samples present some differences in their microwear patterns. That said, it is impossible to propose a rigorous interpretation of these comparisons since too many uncontrolled variables could bias them (sample size, geographical provenience, sex, age, tooth locus and facet analyzed, etc.).

**FIGURE 5:**
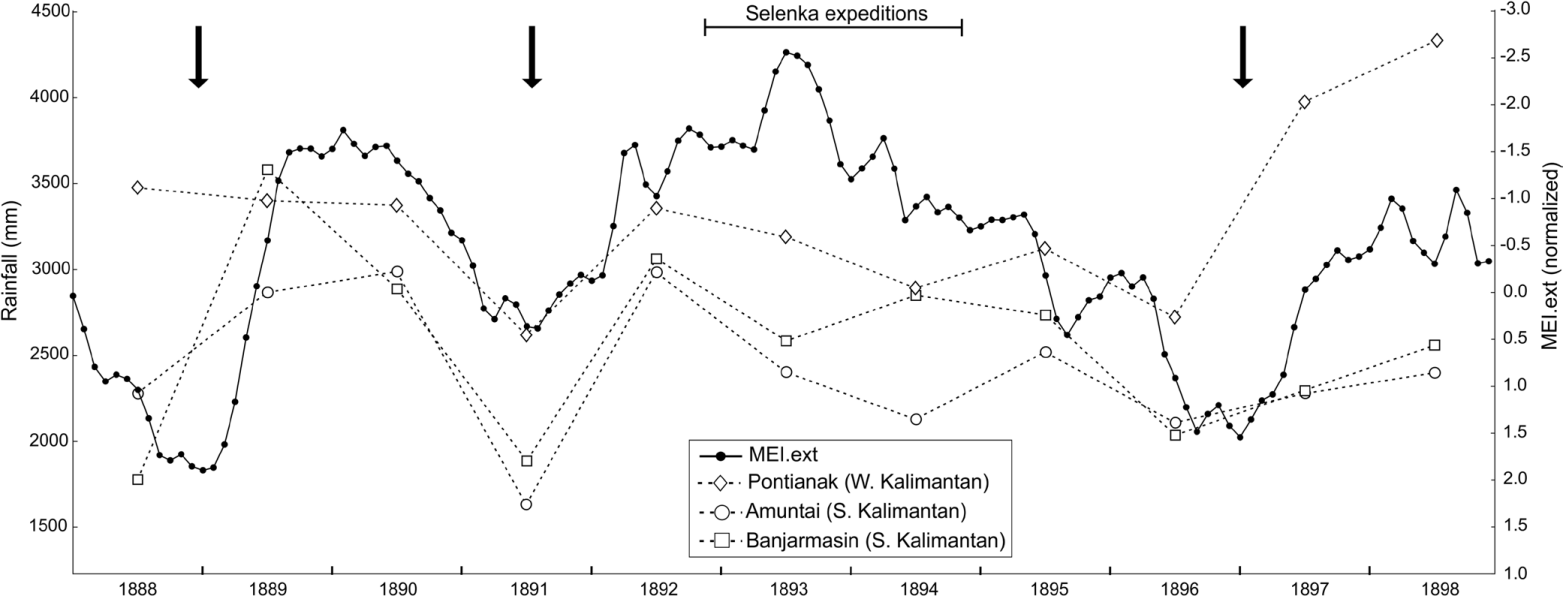
Extended multivariate ENSO index (MEI.ext) and annual rainfall data recorded in three Borneo localities (Pontianak, Amuntai, Banjarmasin) for the decade 1888–1898. Annual precipitation data from Kajita (2019). MEI.ext. data are normalized and represented by standardized departure (see Wolter and Timlin 2011). Arrows mark El Niño events (positive MEI.ext. values; values > |2| indicate strong events). MEI.ext. data from https://psl.noaa.gov/enso/mei.ext/table.ext.html.

More than seasonal effects, mast fruiting events, which occur supra‐annually, have a great impact on orangutan dietary ecology. This effect is more pronounced in Borneo than in Sumatra (Delgado and van Schaik 2000; Knott 1998; Wich et al. 2006). It affects mixed dipterocarp forests, such as those of the study area north of the Kapuas River, more than peat swamp forests (Delgado and van Schaik 2000; Harrison 2010). In masting years, observational studies have shown that the percentage of fruit in these apes' diet may reach 100% (Kanamori et al. 2010). In periods of fruit scarcity, fruit still accounts for at least 12%–20% of the orangutan diet (Kanamori et al. 2010; MacKinnon 1974). For our dataset, it would have been important to assess the probability that the specimens were collected in a mast fruiting year as this would drastically impact their dental microwear textures. Unfortunately, there are no field notes from the Selenka expeditions available where such information could be found. Other reports from contemporaneous zoological expeditions in the same area make no mention of mast fruiting (Büttikofer 1896, 1897; Jentink 1897). However, mast fruiting events have been shown to be linked to the ENSO cycle in a way that the fruiting occurs right at the end of an El Niño event associated with low precipitations (Ashton et al. 1988; Williamson and Ickes 2002; Yasuda et al. 1999). Besides, historical precipitation records from several meteorological stations have been recently published covering the years from 1879 to 1900 (Figure 5; Kajita 2019). One of the three stations from Borneo is located in Pontianak, northeast of the Kapuas River delta. It is the closest one to our study area. The drastic drop in the annual amount of precipitation in 1891 (Kajita 2019) is indeed a good indication for the possibility of a mast fruiting event before the Selenka expedition. No such drop in precipitation is visible for any of the years of the Selenka expeditions (1892–1894).

Another indication comes from the extended Multivariate ENSO index (MEI.ext.; Wolter and Timlin 2011) based on reconstructed sea surface temperatures (Kaplan et al. 1998; Rayner 2003; Smith and Reynolds 2003) and sea level pressures (Allan and Ansell 2006). From 1888 to 1898 this index documents three El Niño events, two strong ones in 1888/1889 and 1896/1897, and a weak one corresponding to the drop in precipitation in 1891 visible in the precipitation records (figure 5; Wolter and Timlin 2011). This index as well, is not consistent with a masting event occurring in one of the years of the Selenka expedition.

Other factors that could influence the dental microwear variation in *Pongo* are age and the sexually differentiated behaviors. Grinding and shearing are two different phases of the mastication process that result in wear facets on different areas of each tooth (Krueger et al. 2008; Maier and Schneck 1981). Thus, a comparison of these two facet types allows for a more comprehensive reconstruction of an animal's diet. It is worth noting that Merceron et al. (2005) did not find any significant differences in dental microwear pattern on both shearing and crushing facets between male and female *Pongo*, or among females depending on gross tooth wear. Using a generalized linear model, Percher et al. (2018) detected effects of sex, social rank, age, and season on dental microwear texture parameters among a wild population of trapped‐and‐released mandrills in Gabon. The absence of a similar record with known life‐history traits for the Borneo population prevents us from applying a glm approach here. Based on the analysis of variances on the principal component scores (Table 3), no significant differences in both sex and age could be detected in the Borneo orangutans. These results advocate for a homogenous diet for the whole sample, at least in regard to the physical properties of the food items consumed. Concerning the age factor, our sample includes specimens with erupted lower second molars, indicating a minimum age of ~5 years (see Smith et al. (1994) for age of dental eruptions in *P. pygmaeus* ). After 5 years, the diet of juvenile *P. pygmaeus* strongly resembles that of the mother (Schuppli et al. 2016) with important dietary‐related mother‐juvenile interactions (Mendonça et al. 2017).

Similarly to our results for the sex and age factors, we found no statistically significant effect of the localities of provenience of the specimens on the DMTA variables. Most localities are from a relatively confined area north of the Kapuas River along both sides of the Ketungau River, except for a single individual from Landak. The fact that Bornean orangutans consume a wide variety of plant taxa might be one factor responsible for this pattern (Russon et al. 2009). If a species focuses its diet on a limited number of taxa thus representing a limited number of physical properties, then changes from this pattern of resource use should be more visible than when many items with different textures and consistencies are consumed as a baseline.

## 5 Conclusion

We used dental microwear textural analysis to assess dietary variation in 89 Bornean orangutans belonging to the late 19th century Selenka collection presently housed at the Zoologische Staatssammlung München (Germany). Using historical precipitation data, an ENSO index, we demonstrated that the Selenka orangutans were probably not collected during a mast fruiting event. Thus, the dietary variation of this orangutan population represents time periods of neutral fruit abundances. Neither sex, nor age or locality from which an individual was collected had a significant influence on their diet or, more precisely, the overall physical properties of the food items consumed in the weeks before its death. Although differences in dietary ecology between sexes were expected due to the results of some observational studies, none were detected in the dental microwear textures of our sample. This study also provides an extensive and important reference dataset for paleodietary reconstructions of extinct hominids as well as purposes in biology conservation for orangutans.

## Author Contributions

**Sophie Gabriele Habinger:** formal analysis, writing – original draft, writing – review and editing, investigation. **Gildas Merceron:** methodology, software, formal analysis, data curation, supervision, conceptualization, investigation, writing – review and editing, writing – original draft. **Anneke H. van Heteren:** investigation, data curation, writing – review and editing. **Valeria Rojas Cuyutupa:** investigation. **Hervé Bocherens:** writing – original draft, writing – review and editing, funding acquisition, conceptualization. **Olivier Chavasseau:** writing – original draft, writing – review and editing, funding acquisition, investigation, formal analysis, conceptualization.

## Funding

This work was supported by Agence Nationale de la Recherche (ANR‐18‐CE92‐0029) and Deutsche Forschungsgemeinschaft (BO 3478/7‐1).

## Supporting information


**Figure S1:** Plots from the PCA. Scree plot (top left), correlation circle (top right), most contributing variables PC1 (middle) and PC2 (bottom). The colors in the correlation circle differentiate between parameters from the two different facets (f6 = red, f9 = blue). The red dashed line indicates the expected contribution per variable, if all of them contributed equally. Thus, variables whose contributions are above this threshold have an important contribution to the PC and were included in the second PCA round.
**Figure S2:** Bivariate plots of PCA scores with individuals categorized by sex. Top: PC1 versus PC2. Bottom: PC1 versus PC3.
**Figure S3:** Boxplots of three SSFA parameters for female (f) and male (m) individuals of the complete data set for crushing and shearing facets.
**Figure S4:** Boxplots of three SSFA parameters for sex‐age groups of all in the data set for crushing and shearing facets. Female (f), male (m), juvenile (juv.), subadult (subad.), and adult (ad.).
**Figure S5:** Boxplots of all individuals in the data set per locality groups.

**Table S1:** Table reporting all the STA and SSFA parameters measured for each individual in the data set including metadata such as about the individuals and the teeth sampled in each case. This table also includes the PCA scores from PC1 to PC4 for each specimen.

## Data Availability

All data generated or analyzed during this study are included in this published article and its Supporting Information files. The data set comprising all individual surface scans (.sur files) is available through the InDoRES data repository (https://www.indores.fr/) with the following https://doi.org/10.48579/PRO/QLGN39.
