# Supplementary material for: Dental Microwear Texture Analysis of the Bornean Orangutans ( Pongo pygmaeus ) From the Selenka Collection
Source: Am J Biol Anthropol. 2026 May 5;190:e70266. doi: 10.1002/ajpa.70266 (PMC13144717; doi:10.1002/ajpa.70266)
Supplement: Supplementary file 1 — Figure S1: Plots from the PCA. Scree plot (top left), correlation circle (top right), most contributing variables PC1 (middle) and PC2 (bottom). The colors in the correlation circle differentiate between parameters from the two different facets (f6 = red, f9 = blue). The red dashed line indicates the expected contribution per variable, if all of them contributed equally. Thus, variables whose contributions are above this threshold have an important contribution to the PC and were included in the second PCA round. Figure S2: Bivariate plots of PCA scores with individuals categorized by sex. Top: PC1 versus PC2. Bottom: PC1 versus PC3. Figure S3: Boxplots of three SSFA parameters for female (f) and male (m) individuals of the complete data set for crushing and shearing facets. Figure S4: Boxplots of three SSFA parameters for sex‐age groups of all in the data set for crushing and shearing facets. Female (f), male (m), juvenile (juv.), subadult (subad.), and adult (ad.). Figure S5: Boxplots of all individuals in the data set per locality groups. [file AJPA-190-e70266-s002.docx]

**Supplementary Information**

Table S1 (provided as a separate file): dataset

History of the *Pongo* Selenka collection	p. 1

Methodology of the dental microwear textural analysis	p. 1

Literature cited	p. 2

Supplementary figures (S1 to S5)	p. 4

Photosimulations and false colour maps of all scans	p. 9

# History of the *Pongo* Selenka collection

Emil Selenka (1842-1902) was a German zoologist who started his scientific career with the study of marine invertebrates before switching his focus to embryology. During his time as professor at the University of Erlangen, he conducted two expeditions to South and Southeast Asia together with his wife Margarethe Lenore Selenka (1860-1922), who studied paleontology, zoology and anthropology. After his arrival in Singapore mid-November 1892, he started a 8-month hunting expedition to Borneo and Sumatra during which he collected orangutan, gibbon and siamang specimens according to documents and correspondence concerning research funding of his expedition by the Königlich Preußischen Akademie der Wissenschaften housed in the Archive of the Berlin-Brandenburgischen Akademie der Wissenschaften (PAW(1812-1945/II-VII-113, The Singapore Free Press and Mercantile Advertiser, 19.11.1892; as well as Selenka & Selenka, 1905). In late 1893, most of the material was lost in shipwreck during transportation on the Kapuas River in West Kalimantan, Borneo (Selenka, 1896). In February 1894, the Selenkas organized a second 10-month hunting expedition led by Margarethe Lenore Selenka as health issues and the nearing end of his leave from university forced Emil Selenka to return to Europe (Hubrecht, 1902-1903; Selenka & Selenka, 1905). He described these events in one of his publications and a letter to his parents written in Medan, Sumatra on February 1^st^ 1894 housed at the University library in Basel (Selenka, 1896; UBH NL 238 : Aa 6,1). The hunters from the second expedition were active in the area on the right bank of the Kapuas River. Here they also traded and purchased orangutan skulls from the local Dajak and Malay.

# Methodology of the dental microwear textural analysis

## **Preparation and scanning of molds**

To remove any dirt or coatings used for preservation, we cleaned the teeth with ethanol using cotton swabs before molding. We then took silicone molds of the teeth (President Regular Body, ref. 6015 - ISO 4823, medium consistency, polyvinylsiloxane addition-type, Coltène-Whaledent). Specific wear facets were scanned with “TRIDENT”, a Leica DCM 8 white-light scanning confocal microscope (Leica Microsystems) with a 100× objective (Numerical aperture = 0.90, Working distance = 0.9 mm) located in the facilities of the PALEVOPRIM laboratory (CNRS and Université de Poitiers, France). We chose facets that represented both phase I shearing facets (f6) and phase II grinding facets (f9) (Maier & Schneck, 1981). Each scan generates a point cloud of 350 × 264 µm. These surfaces were then imported in the LeicaMap v. 8.0 software (Mountain technology, Leica microsystems), and treated following a previously published protocol (Merceron et al. 2016). Then, a 200 × 200 µm grid (1550 × 1550 pixels) was extracted on which the textural parameters are calculated. In cases where there were dirt particles, we shifted the grid to remove these artefacts from the frame. When this was not possible, these spots were smoothened (Laplacian filter) along a manually set border (For the use of Laplacian filter and possible alternatives, see Francisco et al., 2020). Photosimulations and false color maps of all scans used in this study are attached as Supplementary Material.

## **Textural parameters**

Once the surfaces are saved in .sur file format, a second workflow is applied as a template on each surface. It includes a subtraction of a second-order least square polynomial surface (PS2) to remove convexity of the dental facets as it allows for a better visualization of the relief formed by dental microwear (Francisco et al., 2018; Francisco, Brunetière, & Merceron, 2018). Then, a combination of spatial filtering (denoising median 5 × 5 filter size and Gaussian 3 × 3 filter size) and levelling (least square plane by subtraction) is run before extracting surface texture parameters.

Scale Sensitive Fractal Analysis (SSFA) parameters (Scott et al., 2006) were then calculated (see table S1). This analysis is based on a principle from fractal geometry that states that a surface that appears to be smooth on a large scale, can have a rougher more textured profile on a sufficiently fine scale (Scott et al., 2006). We considered complexity, anisotropy and heterogeneity of complexity (Table 2). Complexity (Area-scale fractal complexity – Asfc) is associated with the hardness of food items, with harder items or a larger proportion of harder items in an organism’s diet leading to higher Asfc values. Overlapping pits and scratches of varying sizes and depths characterize these types of highly complex surfaces. Anisotropy (exact proportion Length-scale anisotropy of relief – epLsar) is an indication of food toughness and shows increasing values with structures that share a similar orientation such as parallel striations (Merceron et al., 2005). The heterogeneity of the complexity (Heterogeneity of the Area scale fractal complexity) describes variations of the complexity across a surface. The parameter is calculated on the surface subdivided into a matrix of 3 × 3 (HAsfc9) or 9 × 9 (HAsfc81) sub-surfaces.

Additionally, we also computed Surface Texture Analysis (STA) parameters following well-established procedures (Kaiser, Clauss, & Schulz-Kornas, 2016; Schulz, Calandra, & Kaiser, 2013), as the combination of both SSFA and ISO 25178-2 textural parameters allow for a comprehensive representation of the surface textural information (Calandra et al., 2012). For STA we focused on height parameters (Sq, Ssk, Sku, Sp, Sv, Sz, Sa), surface ones (Sal and Str indexes calculated with z = 0.5 rather than the default setting at 0.2, see Francisco, Brunetière, & Merceron 2018), and volume parameters (metf, medf) parameters (Table 2; Calandra et al., 2012; Kaiser et al., 2016; Schulz et al., 2013).

**Literature cited**

Calandra, I., Schulz, E., Pinnow, M., Krohn, S., & Kaiser, T. M. (2012). Teasing apart the contributions of hard dietary items on 3D dental microtextures in primates. *Journal of Human Evolution*, *63*(1), 85–98. https://doi.org/10.1016/j.jhevol.2012.05.001

Francisco, A., Blondel, C., Brunetière, N., Ramdarshan, A., & Merceron, G. (2018). Enamel surface topography analysis for diet discrimination. A methodology to enhance and select discriminative parameters. *Surface Topography: Metrology and Properties*, *6*(1), 15002. https://doi.org/10.1088/2051-672X/aa9dd3

Francisco, A., Brunetière, N., & Merceron, G. (2018). Gathering and analyzing surface parameters for diet identification purposes. *Technologies*, *6*(3), 75. <https://doi.org/10.3390/technologies6030075>

Francisco, A., Brunetière, N., & Merceron, G. (2020). Damaged digital surfaces also deserve realistic healing. *Surface Topography: Metrology and Properties*, *8*(3), 35008. https://doi.org/10.1088/2051-672X/aba7a3

Hubrecht, A. A. W. (1902-1903). Emil Selenka. In E. Selenka (Ed.), Menschenaffen (Anthropomorphae): Studien über Entwicklung und Schädelbau (pp. 1–14). Wiesbaden: C.W. Kreidel.

Kaiser, T. M., Clauss, M., & Schulz-Kornas, E. (2016). A set of hypotheses on tribology of mammalian herbivore teeth. *Surface Topography: Metrology and Properties*, *4*(1), 14003. https://doi.org/10.1088/2051-672X/4/1/014003

Maier, W., & Schneck, G. (1981). Konstruktionsmorphologische Untersuchungen am Gebiß der hominoiden Primaten. *Zeitschrift Für Morphologie Und Anthropologie*, *72*(2), 127–169. <https://doi.org/10.1127/zma/72/1981/127>

Merceron, G., Blondel, C., Bonis, L. de, Koufos, G. D., & Viriot, L. (2005). A new method of dental microwear analysis: Application to extant primates and *Ouranopithecus macedoniensis* (Late Miocene of Greece). *Palaios*, *20*, 551–561. https://doi.org/10.2110/palo.2004.p04-17

Merceron, G., Ramdarshan, A., Blondel, C., Boisserie, J. R., Brunetiere, N., Francisco, A., … Pret, D. (2016). Untangling the environmental from the dietary: dust does not matter. *Proceedings of the Royal Society B*, *283*(1838), 20161032. <http://dx.doi.org/10.1098/rspb.2016.1032>

Mihlbachler, M. C., Beatty, B. L., Caldera-Siu, A., Chan, D., & Lee, R. (2012). Error rates and observer bias in dental microwear analysis using light microscopy. *Palaeontologia Electronica*, *15*(1), 1–22. <https://doi.org/10.26879/298>

Schulz, E., Calandra, I., & Kaiser, T. M. (2013). Feeding ecology and chewing mechanics in hoofed mammals: 3D tribology of enamel wear. *Wear*, *300*(1-2), 169–179. https://doi.org/10.1016/j.wear.2013.01.115

Scott, R. S., Ungar, P. S., Bergstrom, T. S., Brown, C. A., Childs, B. E., Teaford, M. F., & Walker, A. (2006). Dental microwear texture analysis: technical considerations. *Journal of Human Evolution*, *51(4)*, 339–349. https://doi.org/10.1016/j.jhevol.2006.04.006

Selenka, E. (1896). Die Rassen und der Zahnwechsel des Orang-Utan: vorgelegt von Hrn. Schulze am 5. März. *Sitzungsberichte Der Königlich Preussischen Akademie Der Wissenschaften Zu Berlin*; *16*, 381–392.

Selenka, E., & Selenka, L. (1905). Sonnige Welten: Ostasiatische Reiseskizzen (2nd ed.). Wiesbaden: C.W. Kreidel.

# Results of the principal component analysis

4

Fig. S1. Plots from the PCA. Scree plot (top left), correlation circle (top right), most contributing variables PC1 (middle) and PC2 (bottom). The colors in the correlation circle differentiate between parameters from the two different facets (f6 = red, f9 = blue). The red dashed line indicates the expected contribution per variable, if all of them contributed equally. Thus, variables whose contributions are above this threshold have an important contribution to the PC and were included in the second PCA round.

Fig. S2. Bivariate plots of PCA scores with individuals categorized by sex. Top: PC1 versus PC2. Bottom: PC1 versus PC3.

Fig. S2. Distribution of the individuals along PC1 and PC2 round grouped by sexes.

# Boxplots for SSFA parameters on all groupings and each facet

Fig. S3. Boxplots of three SSFA parameters for female (f) and male (m) individuals of the complete data set for crushing and shearing facets.

Fig. S4. Boxplots of three SSFA parameters for sex-age groups of all in the data set for crushing and shearing facets. Female (f), male (m), juvenile (juv.), subadult (subad.), adult (ad.).

Fig. S5. Boxplots of all individuals in the data set per locality groups.

# Photosimulations and false color maps of all scans

Photosimulations and false color elevation maps of scanned molar phase I and phase II facets of *P. pygmaeus* Borneo (1894).
